# Supplementary material for: Heterogeneity coordinates bacterial multi-gene expression in single cells
Source: PLoS Comput Biol. 2020 Jan 31;16(1):e1007643. doi: 10.1371/journal.pcbi.1007643 (PMC7015429; doi:10.1371/journal.pcbi.1007643)
Supplement: S1 Table — (DOCX) [file pcbi.1007643.s001.docx]

## S1 Table. Sequences of constitutive promoters.

| Names | Sequences |
| --- | --- |
| J23100 | TTGACGGCTAGCTCAGTCCTAGGTACAGTGCTAGC |
| J23119 | TTGACAGCTAGCTCAGTCCTAGGTATAATGCTAGC |
| J23119_L01 | TTGACAGCTAGCTCAGTCCTAGGTATAGAGCTAGC |
| J23119_L09 | TTGACAGCTAGCTCAGTCCTAGGTAAGAAGCTAGC |
| J23119_L16 | TTGACAGCTAGCTCAGTCCTAGGTAGTGTGCTAGC |
| J23119_L18 | TTGACAGCTAGCTCAGTCCTAGGTAGCGTGCTAGC |
| J23119_L19 | TTGACAGCTAGCTCAGTCCTAGGTATATAGCTAGC |
| J23119_L20 | TTGACAGCTAGCTCAGTCCTAGGTAATGTGCTAGC |
