# Supplementary material for: Heterogeneity coordinates bacterial multi-gene expression in single cells
Source: PLoS Comput Biol. 2020 Jan 31;16(1):e1007643. doi: 10.1371/journal.pcbi.1007643 (PMC7015429; doi:10.1371/journal.pcbi.1007643)
Supplement: S2 Table — (DOCX) [file pcbi.1007643.s002.docx]

## S2 Table. Strains used in this study.

| Strains | Descriptions | Genotype |
| --- | --- | --- |
| *E. coli* DH10B | Host cell for operon performance test, negative control in FISH experiments | *Δ(ara-leu) 7697 araD139 fhuA ΔlacX74 galK16 galE15 e14- ϕ80dlacZΔM15 recA1 relA1 endA1 nupG rpsL (StrR) rph spoT1 Δ(mrr-hsdRMS-mcrBC)* |
| *E. coli* DH10GFP | Host cell for sYH006 and sYH013 | *E. coli* DH10B: GFP |
| sYH006 | Used in protein quantification experiments | *E. coli* DH10GFP: pBbA5c-CAR-mCherry |
| sYH013 | Used in mRNA FISH experiments | *E. coli* DH10GFP: pBbA5c-CAR-mCherry(M71G) |
| sYH014 | Operon performance test strain | *E. coli* DH10B: pBbA5c-mCherry-GFP |
| sYH016 | Operon performance test strain | *E. coli* DH10B: pSJ23100c-mCherry-GFP |
| sYH017 | Operon performance test strain | *E. coli* DH10B: pSJ23119c-mCherry-GFP |
| sYHL01 | Operon performance test strain | *E. coli* DH10B: pSJ23119_L01c-mCherry-GFP |
| sYHL09 | Operon performance test strain | *E. coli* DH10B: pSJ23119_L09c-mCherry-GFP |
| sYHL16 | Operon performance test strain | *E. coli* DH10B: pSJ23119_L16c-mCherry-GFP |
| sYHL18 | Operon performance test strain | *E. coli* DH10B: pSJ23119_L18c-mCherry-GFP |
| sYHL19 | Operon performance test strain | *E. coli* DH10B: pSJ23119_L19c-mCherry-GFP |
| sYHL20 | Operon performance test strain | *E. coli* DH10B: pSJ23119_L20c-mCherry-GFP |
