# Supplementary material for: Heterogeneity coordinates bacterial multi-gene expression in single cells
Source: PLoS Comput Biol. 2020 Jan 31;16(1):e1007643. doi: 10.1371/journal.pcbi.1007643 (PMC7015429; doi:10.1371/journal.pcbi.1007643)
Supplement: S3 Table — (DOCX) [file pcbi.1007643.s003.docx]

## S3 Table. Statistics determined by single cell experiments in this work.

1. Normalized single-cell protein abundance. GFP and CAR-mCherry overexpressed from a constitutive and a P_lacUV5_ promoter, respectively in strain sYH006.

| Replicates | IPTG conc. | #cells | Mean | | SD | | CV^2^ | | r |
| --- | --- | --- | --- | --- | --- | --- | --- | --- | --- |
|  |  |  | GFP | mCherry | GFP | mCherry | GFP | mCherry |  |
| A | 0 μm | 2706 | 346.81 | 17.33 | 93.94 | 9.19 | 0.07 | 0.28 | 0.04 |
|  | 12.5 μm | 3321 | 314.42 | 33.54 | 103.78 | 17.54 | 0.11 | 0.27 | 0.31 |
|  | 25 μm | 1882 | 289.31 | 95.13 | 84.57 | 45.36 | 0.09 | 0.23 | 0.36 |
|  | 50 μm | 2665 | 261.57 | 156.59 | 77.18 | 78.91 | 0.09 | 0.25 | 0.42 |
|  | 100 μm | 2449 | 252.36 | 483.54 | 68.71 | 176.61 | 0.07 | 0.13 | 0.50 |
|  | 200 μm | 2483 | 244.37 | 479.11 | 43.81 | 149.79 | 0.03 | 0.10 | 0.28 |
|  | 500 μm | 3047 | 207.51 | 493.65 | 51.99 | 185.82 | 0.06 | 0.14 | 0.52 |
| B | 0 μm | 4918 | 263.95 | 21.03 | 72.19 | 13.24 | 0.07 | 0.40 | 0.14 |
|  | 12.5 μm | 4691 | 344.48 | 44.81 | 89.39 | 21.56 | 0.07 | 0.23 | 0.34 |
|  | 25 μm | 2291 | 271.04 | 65.39 | 69.47 | 32.16 | 0.07 | 0.24 | 0.31 |
|  | 50 μm | 2666 | 264.10 | 157.36 | 72.61 | 77.82 | 0.08 | 0.24 | 0.36 |
|  | 100 μm | 3224 | 264.88 | 470.54 | 92.67 | 177.99 | 0.12 | 0.14 | 0.62 |
|  | 200 μm | 4458 | 245.50 | 627.42 | 64.65 | 231.07 | 0.07 | 0.14 | 0.52 |
|  | 500 μm | 4632 | 179.32 | 555.80 | 44.94 | 198.72 | 0.06 | 0.13 | 0.49 |
| C | 0 μm | 5243 | 326.08 | 12.64 | 109.86 | 5.18 | 0.11 | 0.17 | 0.06 |
|  | 12.5 μm | 2634 | 298.00 | 35.55 | 72.60 | 17.17 | 0.06 | 0.23 | 0.25 |
|  | 25 μm | 2366 | 269.39 | 64.47 | 68.86 | 28.26 | 0.07 | 0.19 | 0.36 |
|  | 50 μm | 4948 | 258.68 | 152.86 | 95.77 | 67.30 | 0.14 | 0.19 | 0.49 |
|  | 100 μm | 1396 | 250.10 | 356.90 | 51.51 | 118.27 | 0.04 | 0.11 | 0.23 |
|  | 200 μm | 3050 | 246.25 | 404.72 | 87.64 | 148.95 | 0.13 | 0.14 | 0.54 |
|  | 500 μm | 1506 | 191.92 | 860.14 | 50.09 | 259.73 | 0.07 | 0.09 | 0.26 |

1. Normalized single-cell protein abundance. GFP and mCherry are overexpressed from the same mRNA using a P_lacUV5_ promoter in strain sYH014.

| Replicates | IPTG conc. | #cells | Mean | | SD | | CV^2^ | | r |
| --- | --- | --- | --- | --- | --- | --- | --- | --- | --- |
|  |  |  | GFP | mCherry | GFP | mCherry | GFP | mCherry |  |
| A | 12.5 μm | 2167 | 37.78 | 28.55 | 20.42 | 15.42 | 0.29 | 0.29 | 0.82 |
|  | 25 μm | 3044 | 157.09 | 102.14 | 98.95 | 64.02 | 0.40 | 0.39 | 0.92 |
|  | 50 μm | 2681 | 460.04 | 261.61 | 163.24 | 96.99 | 0.13 | 0.14 | 0.80 |
|  | 100 μm | 3258 | 783.86 | 450.25 | 207.00 | 118.26 | 0.07 | 0.07 | 0.73 |
|  | 200 μm | 4469 | 764.20 | 454.62 | 182.06 | 115.66 | 0.06 | 0.06 | 0.72 |
|  | 500 μm | 2061 | 785.85 | 400.37 | 238.09 | 139.39 | 0.09 | 0.12 | 0.83 |
| B | 12.5 μm | 741 | 36.36 | 32.89 | 23.24 | 21.84 | 0.41 | 0.44 | 0.90 |
|  | 25 μm | 3801 | 115.95 | 64.45 | 94.44 | 49.37 | 0.66 | 0.59 | 0.85 |
|  | 50 μm | 3688 | 405.65 | 264.46 | 215.97 | 120.00 | 0.28 | 0.21 | 0.87 |
|  | 100 μm | 1538 | 488.11 | 373.91 | 169.15 | 127.18 | 0.12 | 0.12 | 0.85 |
|  | 200 μm | 3001 | 608.40 | 473.28 | 196.42 | 152.88 | 0.10 | 0.10 | 0.85 |
|  | 500 μm | 2015 | 628.64 | 507.50 | 201.07 | 167.81 | 0.10 | 0.11 | 0.87 |
| C | 12.5 μm | 1190 | 32.86 | 35.20 | 18.37 | 17.96 | 0.31 | 0.26 | 0.79 |
|  | 25 μm | 2378 | 162.01 | 109.33 | 94.29 | 63.90 | 0.34 | 0.34 | 0.92 |
|  | 50 μm | 2489 | 460.11 | 361.98 | 186.76 | 136.01 | 0.16 | 0.14 | 0.88 |
|  | 100 μm | 2787 | 619.42 | 339.53 | 254.20 | 158.73 | 0.17 | 0.22 | 0.87 |
|  | 200 μm | 6062 | 562.76 | 436.18 | 199.08 | 152.92 | 0.13 | 0.12 | 0.85 |
|  | 500 μm | 2265 | 754.50 | 462.75 | 317.57 | 147.82 | 0.18 | 0.10 | 0.80 |

1. Normalized single-cell protein abundance. GFP and mCherry are overexpressed from the same mRNA using a library of constitutive promoters with varying strength in strains sYHL01, sYHL09, sYHL16, sYHL18, sYHL19, sYHL20, sYH017, and sYH016.

| Replicates | Library member | #cells | Mean | | SD | | CV^2^ | | r |
| --- | --- | --- | --- | --- | --- | --- | --- | --- | --- |
|  |  |  | GFP | mCherry | GFP | mCherry | GFP | mCherry |  |
| A | L01 | 3261 | 210.02 | 258.62 | 50.11 | 60.87 | 0.06 | 0.06 | 0.74 |
|  | L09 | 3372 | 70.95 | 83.29 | 16.58 | 22.09 | 0.05 | 0.07 | 0.64 |
|  | L16 | 1777 | 812.31 | 1427.82 | 402.30 | 692.25 | 0.25 | 0.24 | 0.96 |
|  | L18 | 1580 | 283.36 | 464.44 | 82.00 | 128.69 | 0.08 | 0.08 | 0.86 |
|  | L19 | 1666 | 944.25 | 964.89 | 295.39 | 468.96 | 0.10 | 0.24 | 0.51 |
|  | L20 | 2044 | 1152.90 | 1820.30 | 453.47 | 754.61 | 0.15 | 0.17 | 0.91 |
|  | J23119 | 3656 | 1521.91 | 1763.20 | 397.18 | 567.23 | 0.07 | 0.10 | 0.61 |
|  | J23100 | 1553 | 1009.44 | 1435.51 | 269.88 | 418.57 | 0.07 | 0.09 | 0.79 |
| B | L01 | 2609 | 223.49 | 327.09 | 63.31 | 87.24 | 0.08 | 0.07 | 0.88 |
|  | L09 | 3280 | 70.94 | 83.56 | 16.53 | 22.07 | 0.05 | 0.07 | 0.67 |
|  | L16 | 2119 | 462.34 | 667.30 | 253.34 | 399.11 | 0.30 | 0.36 | 0.91 |
|  | L18 | 1227 | 282.60 | 466.57 | 81.13 | 128.32 | 0.08 | 0.08 | 0.87 |
|  | L19 | 1811 | 868.06 | 1141.22 | 380.49 | 453.26 | 0.19 | 0.16 | 0.80 |
|  | L20 | 1984 | 1140.50 | 1788.40 | 469.18 | 765.66 | 0.17 | 0.18 | 0.92 |
|  | J23119 | 2363 | 1661.79 | 2359.33 | 489.07 | 748.31 | 0.09 | 0.10 | 0.81 |
|  | J23100 | 1227 | 859.86 | 1246.70 | 193.92 | 338.97 | 0.05 | 0.07 | 0.75 |
| C | L01 | 2572 | 222.15 | 324.11 | 62.87 | 86.49 | 0.08 | 0.07 | 0.87 |
|  | L09 | 4391 | 105.10 | 159.80 | 30.03 | 40.35 | 0.08 | 0.06 | 0.83 |
|  | L16 | 2114 | 462.84 | 659.27 | 258.42 | 398.52 | 0.31 | 0.37 | 0.92 |
|  | L18 | 286 | 291.02 | 563.88 | 76.84 | 157.15 | 0.07 | 0.08 | 0.79 |
|  | L19 | 1804 | 853.94 | 1132.02 | 377.95 | 443.40 | 0.20 | 0.15 | 0.80 |
|  | L20 | 2729 | 873.94 | 1585.14 | 438.60 | 815.68 | 0.25 | 0.26 | 0.95 |
|  | J23119 | 2345 | 1647.28 | 2327.06 | 483.17 | 734.15 | 0.09 | 0.10 | 0.78 |
|  | J23100 | 2200 | 1064.74 | 1501.94 | 285.67 | 434.16 | 0.07 | 0.08 | 0.79 |

1. Single-cell mRNA copy numbers measured from FISH experiments. GFP and CAR-mCherry are transcribed from a constitutive and a P_lacUV5_ promoter, respectively in strain sYH013.

| IPTG conc. | #cells | Mean | | SD | | CV^2^ | | r |
| --- | --- | --- | --- | --- | --- | --- | --- | --- |
|  |  | GFP | mCherry | GFP | mCherry | GFP | mCherry |  |
| 0 μm | 1081 | 1.69 | 0.04 | 1.15 | 0.06 | 0.47 | 2.45 | 0.03 ^a^ |
| 12.5 μm | 1508 | 2.27 | 0.15 | 1.42 | 0.16 | 0.39 | 1.22 | 0.14 ^a^ |
| 25 μm | 975 | 1.96 | 0.43 | 1.33 | 0.42 | 0.46 | 0.95 | 0.23 ^a^ |
| 50 μm | 660 | 2.33 | 3.68 | 1.58 | 3.50 | 0.46 | 0.90 | 0.28 ^a^ |
| 100 μm | 1011 | 1.86 | 4.07 | 1.34 | 3.25 | 0.52 | 0.64 | 0.19 ^a^ |
| 200 μm | 918 | 1.87 | 7.60 | 1.18 | 5.84 | 0.40 | 0.59 | 0.34 ^a^ |
| 500 μm | 721 | 2.27 | 13.84 | 1.57 | 10.28 | 0.48 | 0.55 | 0.18 ^a^ |

SD, standard deviation; CV^2^, squared coefficient of variance; r, Pearson correlation coefficient.

^a^, Correlations were calculated using the raw fluorescence intensity data instead of rounded copy numbers.
