# Supplementary material for: Heterogeneity coordinates bacterial multi-gene expression in single cells
Source: PLoS Comput Biol. 2020 Jan 31;16(1):e1007643. doi: 10.1371/journal.pcbi.1007643 (PMC7015429; doi:10.1371/journal.pcbi.1007643)
Supplement: S4 Table — (DOCX) [file pcbi.1007643.s004.docx]

## S4 Table. Probes used in FISH experiments.

| Probes for sfGFP | | | | Probes for CAR-mCherry | | | |
| --- | --- | --- | --- | --- | --- | --- | --- |
| Probe # | Sequence (5'-> 3') | Position | GC% | Probe # | Sequence (5'-> 3') | Position | GC% |
| 1 | tgaacagttcttcgccttta | 6 | 40.00% | 1 | catgttatcctcctcgcc | 13 | 56.00% |
| 2 | gaccagaatcggaactacgc | 29 | 55.00% | 2 | gcatgaactccttgatga | 35 | 44.00% |
| 3 | acttatgaccgttcacatcg | 60 | 45.00% | 3 | ctccatgtgcaccttgaa | 55 | 50.00% |
| 4 | tggtgcagatgaacttcagg | 129 | 50.00% | 4 | cctcgatctcgaactcgt | 89 | 56.00% |
| 5 | tacgtcaacgtcgtcaccaa | 178 | 50.00% | 5 | ttggtcaccttcagcttg | 147 | 50.00% |
| 6 | aacgcgcaaaacactgcacg | 201 | 55.00% | 6 | aggatgtcccaggcgaag | 180 | 61.00% |
| 7 | tcgtgttgtttcatgtggtc | 226 | 45.00% | 7 | gccgtacatgaactgagg | 202 | 56.00% |
| 8 | cggcatcgcagatttgaaga | 248 | 50.00% | 8 | tgcttcacgtaggccttg | 222 | 56.00% |
| 9 | aaggaaatggtacgctcctg | 280 | 50.00% | 9 | agtagtcggggatgtcgg | 245 | 61.00% |
| 10 | gttttgtagtagccatcatc | 304 | 40.00% | 10 | ctcggggaaggacagctt | 265 | 61.00% |
| 11 | ttcaaacttaacctctgcgc | 326 | 45.00% | 11 | acgcgctcccacttgaag | 285 | 61.00% |
| 12 | ttcgatacgattgaccagcg | 353 | 50.00% | 12 | cgccgtcctcgaagttca | 305 | 61.00% |
| 13 | ctctttaaagtcgataccct | 377 | 40.00% | 13 | gaggagtcctgggtcacg | 333 | 67.00% |
| 14 | tttatggcccagaatgttac | 401 | 40.00% | 14 | tagatgaactcgccgtcc | 357 | 56.00% |
| 15 | ggctgttgaagttatactcc | 423 | 45.00% | 15 | gaagttggtgccgcgcag | 385 | 67.00% |
| 16 | cttgtctgccgtaatgtaaa | 449 | 40.00% | 16 | attacggggccgtcggag | 405 | 67.00% |
| 17 | tgaaattggccttgatgccg | 477 | 50.00% | 17 | cagcccatggtcttcttc | 426 | 56.00% |
| 18 | tcctcaacattgtggcgaat | 499 | 45.00% | 18 | tacatccgctcggaggag | 450 | 61.00% |
| 19 | tgttctgctggtaatggtcg | 537 | 50.00% | 19 | tgatctcgcccttcaggg | 482 | 61.00% |
| 20 | aaaaccggaccgtcaccaat | 562 | 50.00% | 20 | cttcagcttcagcctctg | 502 | 56.00% |
| 21 | gctcagatagtgattatccg | 587 | 45.00% | 21 | ttgacctcagcgtcgtag | 531 | 56.00% |
| 22 | tctttgctcagcacgctttg | 610 | 50.00% | 22 | ttcttggccttgtaggtg | 552 | 50.00% |
| 23 | gtgatcacgtttttcgttcg | 632 | 45.00% | 23 | tgttgacgttgtaggcgc | 587 | 56.00% |
| 24 | gtcacaaattccagcaggac | 655 | 50.00% | 24 | gggaggtgatgtccaact | 608 | 56.00% |
| 25 | cttatacagctcgtccatac | 695 | 45.00% | 25 | gatggtgtagtcctcgtt | 628 | 50.00% |
| 26 | agcgcgtaattctcatcatt | 727 | 40.00% | 26 | ggcgcgttcgtactgttc | 649 | 61.00% |
|  |  |  |  | 27 | cttgtacagctcgtccat | 691 | 50.00% |
