# Supplementary material for: Heterogeneity coordinates bacterial multi-gene expression in single cells
Source: PLoS Comput Biol. 2020 Jan 31;16(1):e1007643. doi: 10.1371/journal.pcbi.1007643 (PMC7015429; doi:10.1371/journal.pcbi.1007643)
Supplement: S1 Text — (DOCX) [file pcbi.1007643.s005.DOCX]

**Heterogeneity coordinates bacterial multi-gene expression in single cells**

**Authors:** Yichao Han^1^, Fuzhong Zhang^1-3,^ *

Affiliations:

^1^Department of Energy, Environmental and Chemical Engineering,

^2^ Division of Biological & Biomedical Sciences,

^3^ Institute of Materials Science & Engineering,

Washington University in St. Louis, St. Louis, MO 63130, USA.

*e-mail: [fzhang@seas.wustl.edu](mailto:fzhang@seas.wustl.edu)

**S1 Text. Models and Parameters**

# Note 1. Translational resource competition model

## Note 1.1 Model description

We assume that ribosomes exemplify the only limited resource during translation. There are three steps in the translational resource competition model: 1) formation of mRNA-ribosome complexes, 2) generation of proteins from mRNA-ribosome complexes, and 3) degradation of proteins (Fig 2A).

|  | $M_{i}^{F}+n_{i} {Rib}^{F} \underset{\to}{\beta_{i}^{+}}M_{i}^{C}$ $M_{i}^{C} \underset{\to}{\beta_{i}^{-}}M_{i}^{F}+n_{i} {Rib}^{F}+n_{i}P_{i}$ $P_{i} \underset{\to}{\lambda}\emptyset$ | (1) |
| --- | --- | --- |

We consider that multiple ribosome molecules ($n_{i}$) can simultaneously translate the same mRNA. For either heterologous mRNA (*i*=1) or endogenous mRNA (*i*=2), free mRNA ($M_{i}^{F}$) is bound by $n_{i}$free ribosomes (${Rib}^{F}$), forming mRNA-ribosome complex ($M_{i}^{C}$) at rate $\beta_{i}^{+}$, and then generates $n_{i}$ proteins ($P_{i}$) at rate $\beta_{i}^{+}$. Protein degradation is negligible compared to protein dilution due to cell growth, thus the rate $\lambda$ is equivalent to the growth rate.

The dynamics of ${Rib}^{F}$, $M_{i}^{C}$, and $P_{i}$ are described by

|  | $\frac{d{Rib}^{F}}{dt}=\sum_{i=1}^{2} n_{i} (\beta_{i}^{-} M_{i}^{C}-\beta_{i}^{+} M_{i}^{F} {Rib}^{F})$ | (2) |
| --- | --- | --- |
|  | $\frac{dM_{i}^{C}}{dt}=\beta_{i}^{+} M_{i}^{F} {Rib}^{F}-\beta_{i}^{-} M_{i}^{C}$ | (3) |
|  | $\frac{dP_{i}}{dt}=n_{i} \beta_{i}^{-} M_{i}^{C}-\lambda P_{i}.$ | (4) |

The total ribosomes $({Rib}^{T})$ and total mRNAs ($M_{i}^{T}$) inside a cell are treated as constants during transcription resource allocation, giving two constraints through mass balance:

|  | ${Rib}^{F}+\sum_{i=1}^{2} n_{i}\cdot M_{i}^{C}={Rib}^{T}$ | (5) |
| --- | --- | --- |
|  | $M_{i}^{F}+M_{i}^{C}=M_{i}^{T}, i=1,2.$ | (6) |

## Note 1.2 Solving for steady states

Equations (2)-(4) can be solved for steady state by setting all differentials as zeros. The steady state abundances of the mRNA-ribosome complex ($M_{i}^{C}$) and the corresponding proteins ($P_{i}$) are given by

|  | $M_{i}^{C}=\frac{\beta_{i}^{+} {Rib}^{F} M_{i}^{F}}{\beta_{i}^{-}}$ | (7) |
| --- | --- | --- |
|  | $P_{i}=\frac{n_{i} \beta_{i}^{-} M_{i}^{C}}{\lambda}.$ | (8) |

Combining equations (6) and (7), we obtain

|  | $M_{i}^{C}=\frac{{Rib}^{F}}{\beta_{i}+{Rib}^{F}}M_{i}^{T},$ | (9) |
| --- | --- | --- |

where $\beta_{i}=\beta_{i}^{-}/\beta_{i}^{+}$ is the dissociation constant.

Substituting equation (9) into (5), we have

|  | ${Rib}^{F}+\frac{n_{1}{Rib}^{F}}{\beta_{1}+{Rib}^{F}}M_{1}^{T}+\frac{n_{2}{Rib}^{F}}{\beta_{2}+{Rib}^{F}}M_{2}^{T}={Rib}^{T}.$ | (10) |
| --- | --- | --- |

Given $M_{1}^{T}$, $M_{2}^{T}$, and ${Rib}^{T}$, the steady state ${Rib}^{F}$ can be calculated by solving equation (10). Combining (7), (8), and (10), $P_{i}$ at steady state for *i*=1, 2 are expressed in terms of ${Rib}^{F}$:

|  | $P_{i}=\frac{n_{i}\cdot\beta_{i}^{-}}{\lambda}\cdot\frac{{Rib}^{F}}{\beta_{i}+{Rib}^{F}}{\cdot M}_{i}^{T}.$ | (11) |
| --- | --- | --- |

## Note 1.3 Introducing variations

Although we assume that the total ribosomes $({Rib}^{T})$ and total mRNAs ($M_{i}^{T}$) inside a cell are treated as constant, $M_{1}^{T}$, $M_{2}^{T}$, and ${Rib}^{T}$ are considered as random variables of a population of cells. How cell-to-cell variations of $M_{1}^{T}$, $M_{2}^{T}$, and ${Rib}^{T}$ affect cell-to-cell variations of $P_{i}$ is evaluated by using the linear approximation

|  | $\left( \begin{matrix} P_{1} \\ P_{2} \end{matrix} \right)=\left( \begin{matrix} \bar{P_{1}} \\ \bar{P_{2}} \end{matrix} \right)+\Psi\left( \begin{matrix} M_{1}^{T}-\bar{M_{1}^{T}} \\ M_{2}^{T}-\bar{M_{2}^{T}} \\ {Rib}^{T}-\bar{{Rib}^{T}} \end{matrix} \right),$ | (12) |
| --- | --- | --- |

where $\bar{X}$ denotes the mean value of $X$ at steady state, and $\Psi=\binom{\begin{matrix} \psi_{11} & \psi_{12} & \psi_{13} \end{matrix}}{\begin{matrix} \psi_{21} & \psi_{22} & \psi_{23} \end{matrix}}=\binom{\begin{matrix} \frac{\partial P_{1}}{\partial M_{1}^{T}} & \frac{\partial P_{1}}{\partial M_{2}^{T}} & \frac{\partial P_{1}}{\partial{Rib}^{T}} \end{matrix}}{\begin{matrix} \frac{\partial P_{2}}{\partial M_{1}^{T}} & \frac{\partial P_{2}}{\partial M_{2}^{T}} & \frac{\partial P_{2}}{\partial{Rib}^{T}} \end{matrix}}$ is the Jacobian matrix at steady state.

Then the variance and covariance of $P_{1}$ and $P_{2}$ can be derived as

|  | $Var\left( P_{1} \right)={\psi_{11}}^{2}Var\left( M_{1}^{T} \right)+{\psi_{12}}^{2}Var\left( M_{2}^{T} \right)+{\psi_{13}}^{2}Var\left( {Rib}^{T} \right)+2\psi_{11}\psi_{12}Cov\left( M_{1}^{T},M_{2}^{T} \right)+2\psi_{11}\psi_{13}Cov\left( M_{1}^{T},{Rib}^{T} \right)+2\psi_{12}\psi_{13}Cov\left( M_{2}^{T},{Rib}^{T} \right)$ | (13) |
| --- | --- | --- |
|  | $Var(P_{2})={\psi_{21}}^{2}Var\left( M_{1}^{T} \right)+{\psi_{22}}^{2}Var\left( M_{2}^{T} \right)+{\psi_{23}}^{2}Var\left( {Rib}^{T} \right)+2\psi_{21}\psi_{22}Cov\left( M_{1}^{T},M_{2}^{T} \right)+2\psi_{21}\psi_{23}Cov\left( M_{1}^{T},{Rib}^{T} \right)+2\psi_{22}\psi_{23}Cov\left( M_{2}^{T},{Rib}^{T} \right)$ | (14) |
|  | $Cov\left( P_{1},P_{2} \right)=\psi_{11}\psi_{21}Var\left( M_{1}^{T} \right)+\psi_{12}\psi_{22}Var\left( M_{2}^{T} \right)+\psi_{13}\psi_{23}Var\left( {Rib}^{T} \right)$ $+\left( \psi_{11}\psi_{22}+\psi_{21}\psi_{12} \right)Cov\left( M_{1}^{T},M_{2}^{T} \right)+\left( \psi_{11}\psi_{23}+\psi_{21}\psi_{13} \right)Cov\left( M_{1}^{T},{Rib}^{T} \right)+\left( \psi_{23}\psi_{12}+\psi_{13}\psi_{22} \right)Cov\left( M_{2}^{T},{Rib}^{T} \right).$ | (15) |

To obtain the Jacobian matrix $\Psi$, we first analyze $\frac{\partial{Rib}^{F}}{\partial M_{1}^{T}}$, $\frac{\partial{Rib}^{F}}{\partial M_{2}^{T}}$, and $\frac{\partial{Rib}^{F}}{\partial{Rib}^{T}}$. We differentiate both sides of equation (10) with respect to $M_{1}^{T}$, $M_{2}^{T}$, and ${Rib}^{T}$, respectively, and obtain

|  | $\frac{\partial{Rib}^{F}}{\partial M_{1}^{T}}+\frac{n_{1} {Rib}^{F}}{\beta_{1}+{Rib}^{F}}+\sum_{i=1}^{2} \frac{n_{i}\beta_{i}M_{i}^{T}}{\left( \beta_{i}+{Rib}^{F} \right)^{2}}\cdot\frac{\partial{Rib}^{F}}{\partial M_{1}^{T}}=0$ | (16) |
| --- | --- | --- |
|  | $\frac{\partial{Rib}^{F}}{\partial M_{2}^{T}}+\frac{n_{2} {Rib}^{F}}{\beta_{2}+{Rib}^{F}}+\sum_{i=1}^{2} \frac{n_{i}\beta_{i}M_{i}^{T}}{\left( \beta_{i}+{Rib}^{F} \right)^{2}}\cdot\frac{\partial{Rib}^{F}}{\partial M_{2}^{T}}=0$ | (17) |
|  | $\frac{\partial{Rib}^{F}}{\partial{Rib}^{T}}(1+\frac{n_{1}\beta_{1}M_{1}^{T}}{\left( \beta_{1}+{Rib}^{F} \right)^{2}}+\frac{n_{2}\beta_{2}M_{2}^{T}}{\left( \beta_{2}+{Rib}^{F} \right)^{2}})=1.$ | (18) |

The expressions and the signs of $\frac{\partial{Rib}^{F}}{\partial M_{1}^{T}}$, $\frac{\partial{Rib}^{F}}{\partial M_{2}^{T}}$, and $\frac{\partial{Rib}^{F}}{\partial{Rib}^{T}}$ are determined as follows:

|  | $\frac{\partial{Rib}^{F}}{\partial M_{1}^{T}}=-\frac{\frac{n_{1} {Rib}^{F}}{\beta_{1}+{Rib}^{F}}}{1+\sum_{i=1}^{2} \frac{n_{i}\beta_{i}M_{i}^{T}}{\left( \beta_{i}+{Rib}^{F} \right)^{2}}}<0$ | (19) |
| --- | --- | --- |
|  | $\frac{\partial{Rib}^{F}}{\partial M_{2}^{T}}=-\frac{\frac{n_{2} {Rib}^{F}}{\beta_{2}+{Rib}^{F}}}{1+\sum_{i=1}^{2} \frac{n_{i}\beta_{i}M_{i}^{T}}{\left( \beta_{i}+{Rib}^{F} \right)^{2}}}<0$ | (20) |
|  | $\frac{\partial{Rib}^{F}}{\partial{Rib}^{T}}=\frac{1}{1+\frac{n_{1}\beta_{1}M_{1}^{T}}{\left( \beta_{1}+{Rib}^{F} \right)^{2}}+\frac{n_{2}\beta_{2}M_{2}^{T}}{\left( \beta_{2}+{Rib}^{F} \right)^{2}}}>0.$ | (21) |

Reorganizing equations (16)-(18) yields

|  | $\frac{n_{1}\beta_{1}M_{1}^{T}}{\left( \beta_{1}+{Rib}^{F} \right)^{2}}\cdot\frac{\partial{Rib}^{F}}{\partial M_{1}^{T}}+\frac{n_{1} {Rib}^{F}}{\beta_{1}+{Rib}^{F}}=-\left( 1+\frac{n_{2}\beta_{2}M_{2}^{T}}{\left( \beta_{2}+{Rib}^{F} \right)^{2}} \right)\frac{\partial{Rib}^{F}}{\partial M_{1}^{T}}>0$ | (22) |
| --- | --- | --- |
|  | $\frac{n_{2}\beta_{2}M_{2}^{T}}{\left( \beta_{2}+{Rib}^{F} \right)^{2}}\cdot\frac{\partial{Rib}^{F}}{\partial M_{2}^{T}}+\frac{n_{2} {Rib}^{F}}{\beta_{2}+{Rib}^{F}}=-\left( 1+\frac{n_{1}\beta_{1}M_{1}^{T}}{\left( \beta_{1}+{Rib}^{F} \right)^{2}} \right)\frac{\partial{Rib}^{F}}{\partial M_{2}^{T}}>0.$ | (23) |

Then the elements in the Jacobian matrix $\Psi$ are further derived in terms of $\frac{\partial{Rib}^{F}}{\partial M_{1}^{T}}$, $\frac{\partial{Rib}^{F}}{\partial M_{2}^{T}}$, and $\frac{\partial{Rib}^{F}}{\partial{Rib}^{T}}$:

|  | $\psi_{11}=\frac{\partial P_{1}}{\partial M_{1}^{T}}=\frac{n_{1} \beta_{1}^{-}}{\lambda}\cdot\left( \frac{\beta_{1} M_{1}^{T}}{\left( \beta_{1}+{Rib}^{F} \right)^{2}}\cdot\frac{\partial{Rib}^{F}}{\partial M_{1}^{T}}+\frac{{Rib}^{F}}{\beta_{1}+{Rib}^{F}} \right)>0$ | (24) |
| --- | --- | --- |
|  | $\psi_{21}=\frac{\partial P_{2}}{\partial M_{1}^{T}}=\frac{n_{2} \beta_{2}^{-}}{\lambda}\cdot\frac{\beta_{2} M_{2}^{T}}{\left( \beta_{2}+{Rib}^{F} \right)^{2}}\cdot\frac{\partial{Rib}^{F}}{\partial M_{1}^{T}}<0$ | (25) |
|  | $\psi_{12}=\frac{\partial P_{1}}{\partial M_{2}^{T}}=\frac{n_{1} \beta_{1}^{-}}{\lambda}\cdot\frac{\beta_{1} M_{1}^{T}}{\left( \beta_{1}+{Rib}^{F} \right)^{2}}\cdot\frac{\partial{Rib}^{F}}{\partial M_{2}^{T}}<0$ | (26) |
|  | $\psi_{22}=\frac{\partial P_{2}}{\partial M_{2}^{T}}=\frac{n_{2} \beta_{2}^{-}}{\lambda}\cdot\left( \frac{\beta_{2} M_{2}^{T}}{\left( \beta_{2}+{Rib}^{F} \right)^{2}}\cdot\frac{\partial{Rib}^{F}}{\partial M_{2}^{T}}+\frac{{Rib}^{F}}{\beta_{2}+{Rib}^{F}} \right)>0$ | (27) |
|  | $\psi_{13}=\frac{\partial P_{1}}{\partial{Rib}^{T}}=\frac{n_{1} \beta_{1}^{-}}{\lambda}\cdot\frac{\beta_{1} M_{1}^{T}}{\left( \beta_{1}+{Rib}^{F} \right)^{2}}\cdot\frac{\partial{Rib}^{F}}{\partial{Rib}^{T}}>0$ | (28) |
|  | $\psi_{23}=\frac{\partial P_{2}}{\partial{Rib}^{T}}=\frac{n_{2} \beta_{2}^{-}}{\lambda}\cdot\frac{\beta_{2} M_{2}^{T}}{\left( \beta_{2}+{Rib}^{F} \right)^{2}}\cdot\frac{\partial{Rib}^{F}}{\partial{Rib}^{T}}>0,$ | (29) |

The signs of coefficients for $Var\left( M_{1}^{T} \right)$, $Var\left( M_{2}^{T} \right)$, $Var\left( {Rib}^{T} \right)$, and $Cov\left( M_{1}^{T},M_{2}^{T} \right)$ in equation (15) are determined as follow:

|  | $\psi_{11}\psi_{21}<0$ | (30) |
| --- | --- | --- |
|  | $\psi_{12}\psi_{22}<0$ | (31) |
|  | $\psi_{13}\psi_{23}>0$ | (32) |
|  | $\psi_{11}\psi_{22}+\psi_{21}\psi_{12}>0.$ | (33) |

The coefficient for $Cov\left( M_{1}^{T},{Rib}^{T} \right)$ is

|  | $\psi_{11}\psi_{23}+\psi_{21}\psi_{13}=\frac{n_{1} \beta_{1}^{-}n_{2} \beta_{2}^{-}M_{1}^{T}\beta_{2} M_{2}^{T}}{\lambda^{2}\left( \beta_{1}+{Rib}^{F} \right)\left( \beta_{2}+{Rib}^{F} \right)^{2}}\frac{\partial{Rib}^{F}}{\partial{Rib}^{T}}$ ($\frac{2\beta_{1}}{\beta_{1}+{Rib}^{F}}\cdot\frac{\partial{Rib}^{F}}{\partial M_{1}^{T}}+\frac{{Rib}^{F}}{M_{1}^{T}}).$ | (34) |
| --- | --- | --- |

The sign of $\psi_{11}\psi_{23}+\psi_{21}\psi_{13}$ is the same as the sign of$\frac{2\beta_{1}}{\beta_{1}+{Rib}^{F}}\cdot\frac{\partial{Rib}^{F}}{\partial M_{1}^{T}}+\frac{{Rib}^{F}}{M_{1}^{T}}$. We first notice $-\frac{\partial{Rib}^{F}}{\partial M_{1}^{T}}<\frac{n_{1} {Rib}^{F}}{\beta_{1}+{Rib}^{F}}$ from equation (19). Then an upper bound of $-\frac{2\beta_{1}}{\beta_{1}+{Rib}^{F}}\cdot\frac{\partial{Rib}^{F}}{\partial M_{1}^{T}}$ can be obtained through the inequality $-\frac{2\beta_{1}}{\beta_{1}+{Rib}^{F}}\cdot\frac{\partial{Rib}^{F}}{\partial M_{1}^{T}}<\frac{2n_{1}\beta_{1}{Rib}^{F}}{\left( \beta_{1}+{Rib}^{F} \right)^{2}}<\frac{n_{1}}{2}$. Although this upper bound is not tight enough, $\frac{2\beta_{1}}{\beta_{1}+{Rib}^{F}}\cdot\frac{\partial{Rib}^{F}}{\partial M_{1}^{T}}+\frac{{Rib}^{F}}{M_{1}^{T}}$ is positive at least when $n_{1}M_{1}^{T}$ is smaller than $2{Rib}^{F}$, which is generally true for most heterologous expression. Therefore, $\psi_{11}\psi_{23}+\psi_{21}\psi_{13}$is positive. Similarly, $\psi_{23}\psi_{12}+\psi_{13}\psi_{22}$ (the coefficient for $Cov\left( M_{2}^{T},{Rib}^{T} \right)$) is also positive. Collectively, $Var\left( M_{1}^{T} \right)$ and $Var\left( M_{2}^{T} \right)$ contribute negatively to the covariance $Cov\left( P_{1},P_{2} \right)$, while $Var\left( {Rib}^{T} \right)$, $Cov\left( M_{1}^{T},M_{2}^{T} \right)$, $Cov\left( M_{1}^{T},{Rib}^{T} \right)$ and $Cov\left( M_{2}^{T},{Rib}^{T} \right)$contribute positively to the covariance $Cov\left( P_{1},P_{2} \right)$. Among these, the variances of $M_{1}^{T}$ and ${Rib}^{T}$ are major factors that make opposite contributions. For simplicity, we focus on these two factors and neglect other terms in equations (13)-(15):

| $Var\left( P_{1} \right)={(\frac{\partial P_{1}}{\partial M_{1}^{T}})}^{2}Var\left( M_{1}^{T} \right)+{(\frac{\partial P_{1}}{\partial{Rib}^{T}})}^{2}Var\left( {Rib}^{T} \right)$ | (35) |
| --- | --- |
| $Var\left( P_{2} \right)=\left( \frac{\partial P_{2}}{\partial M_{1}^{T}} \right)^{2}Var\left( M_{1}^{T} \right)+\left( \frac{\partial P_{2}}{\partial{Rib}^{T}} \right)^{2}Var\left( {Rib}^{T} \right)$ | (36) |
| $Cov\left( P_{1},P_{2} \right)=\frac{\partial P_{1}}{\partial M_{1}^{T}}\frac{\partial P_{2}}{\partial M_{1}^{T}}Var\left( M_{1}^{T} \right)+\frac{\partial P_{1}}{\partial{Rib}^{T}}\frac{\partial P_{2}}{\partial{Rib}^{T}}Var\left( {Rib}^{T} \right).$ | (37) |

Equation (37) reproduces equation (1) in the main text to explain the effects of resource competition and heterogeneity.

Using biologically feasible parameters (Table A), the Pearson correlation coefficients ($r=\frac{Cov\left( P_{1},P_{2} \right)}{\sqrt{Var\left( P_{1} \right)\cdot Var\left( P_{2} \right)}}$) are calculated at various means and variances of both $M_{1}^{T}$ and ${Rib}^{T}$, showing that both positive and negative correlations are achievable by merely tuning the properties of these two random variables (Fig 2 C-F and S2C-J Fig).

# Note 2. Transcription resource competition model

## Note 2.1 Model description

We extend the modeling framework used in Supplementary Note 1 and apply it to transcription resource competition, where three steps are involved: 1) formation of the gene-RNAP complex, 2) generation of RNA from the gene-RNAP complex, and 3) degradation of RNA.

|  | $D_{i}^{F}+\nu_{i} {RNAP}^{F}\underset{\to}{\alpha_{i}^{+}}D_{i}^{C}$ $D_{i}^{C} \underset{\to}{\alpha_{i}^{-}}D_{i}^{F}+\nu_{i} {RNAP}^{F}+{\nu_{i} M}_{i}$ $M_{i} \underset{\to}{\gamma_{i}}\emptyset$ | (38) |
| --- | --- | --- |

In this model, $D_{i}^{F}$ represents the numbers of empty promoters of heterologous genes (*i=1*), endogenous mRNA-coding genes (*i=2*), or rRNA and tRNA genes (*i=3*). In addition, $D_{i}^{C}$, ${RNAP}^{F}$, and $M_{i}$respectively represent the RNAP-gene complex, free RNAPs, and RNA. We assume multiple RNAPs can transcribe the same gene simultaneously [1]. The parameter $\nu_{i}$ is used to describe the number of RNAPs simultaneously transcribing the same gene. The parameters $\alpha_{i}^{+}$and $\alpha_{i}^{-}$ describe the rates of the first two steps. For RNA degradation, RNAs with stable secondary structures (e.g., tRNAs and rRNAs) have a slow degradation rate $\gamma_{3}$, which is close to the growth rate; conversely, mRNAs usually degrade rapidly, with $\gamma_{1}$ and $\gamma_{2}$ at min^-1^ scale [2]. Regulation of transcriptional factors is not considered in this model, although transcriptional factors can be analyzed as resources using the modeling framework.

The model is described by the following differential and conservation equations:

|  | $\frac{d{RNAP}^{F}}{dt}=\sum_{i=1}^{3} \nu_{i}(\alpha_{i}^{-} D_{i}^{C}-\alpha_{i}^{+} D_{i}^{F} {RNAP}^{F})$ | (39) |
| --- | --- | --- |
|  | $\frac{dD_{i}^{C}}{dt}=\alpha_{i}^{+} D_{i}^{F} {RNAP}^{F}-\alpha_{i}^{-} D_{i}^{C}$ | (40) |
|  | $\frac{dM_{i}}{dt}=\nu_{i} \alpha_{i}^{-} D_{i}^{C}-\gamma_{i} M_{i}$ | (41) |
|  | ${RNAP}^{F}+\sum_{i=1}^{3} \nu_{i} D_{i}^{C}={RNAP}^{T}$ | (42) |
|  | $D_{i}^{F}+D_{i}^{C}=D_{i}^{T}.$ | (43) |

## Note 2.2 Solving for steady states

Similar to equation (9), we obtain

|  | $D_{i}^{C}=\frac{{RNAP}^{F}}{\alpha_{i}+{RNAP}^{F}}D_{i}^{T},$ | (44) |
| --- | --- | --- |

where $\alpha_{i}=\alpha_{i}^{-}/\alpha_{i}^{+}$ is the dissociation constant for RNAP binding.

Substituting equation (44) into (42), we have

|  | ${RNAP}^{F}+\frac{\nu_{1} {RNAP}^{F}}{\alpha_{1}+{RNAP}^{F}}D_{1}^{T}+\frac{\nu_{2} {RNAP}^{F}}{\alpha_{2}+{RNAP}^{F}}D_{2}^{T}+\frac{\nu_{3} {RNAP}^{F}}{\alpha_{3}+{RNAP}^{F}}D_{3}^{T}={RNAP}^{T}.$ | (45) |
| --- | --- | --- |

The steady state of ${RNAP}^{F}$ can be calculated by solving equation (45) when $D_{i}^{T}$ (*i*=1, 2, 3) and ${RNAP}^{T}$ are given. Then $M_{i}$ can be calculated in terms of ${RNAP}^{F}$:

|  | $M_{i}=\frac{\nu_{i} \alpha_{i}^{-}}{\gamma_{i}}\cdot\frac{{RNAP}^{F}}{\alpha_{i}+{RNAP}^{F}}\cdot D_{i}^{T}.$ | (46) |
| --- | --- | --- |

The same method of analyzing correlation used in Supplementary Note 1 is also applied here. Similar to equations (35)-(37), the variances and covariance of $M_{1}$ and $M_{2}$ are given by

|  | $Var\left( M_{1} \right)={\frac{\partial M_{1}}{\partial D_{1}^{T}}}^{2}Var\left( D_{1}^{T} \right)+{\frac{\partial M_{1}}{\partial D_{2}^{T}}}^{2}Var\left( D_{2}^{T} \right)+{\frac{\partial M_{1}}{\partial{RNAP}^{T}}}^{2}Var\left( {RNAP}^{T} \right)+2\frac{\partial M_{1}}{\partial D_{1}^{T}}\frac{\partial M_{1}}{\partial D_{2}^{T}}Cov\left( D_{1}^{T},D_{2}^{T} \right)+2\frac{\partial M_{1}}{\partial D_{1}^{T}}\frac{\partial M_{1}}{\partial{RNAP}^{T}}Cov\left( D_{1}^{T},{RNAP}^{T} \right)+2\frac{\partial M_{1}}{\partial D_{2}^{T}}\frac{\partial M_{1}}{\partial{RNAP}^{T}}Cov\left( D_{2}^{T},{RNAP}^{T} \right)$ | (47) |
| --- | --- | --- |
|  | $Var(M_{2})={\frac{\partial M_{2}}{\partial D_{1}^{T}}}^{2}Var\left( D_{1}^{T} \right)+{\frac{\partial M_{2}}{\partial D_{2}^{T}}}^{2}Var\left( D_{2}^{T} \right)+{\frac{\partial M_{2}}{\partial{RNAP}^{T}}}^{2}Var({RNAP}^{T})+2\frac{\partial M_{2}}{\partial D_{1}^{T}}\frac{\partial M_{2}}{\partial D_{2}^{T}}Cov\left( D_{1}^{T},D_{2}^{T} \right)+2\frac{\partial M_{2}}{\partial D_{1}^{T}}\frac{\partial M_{2}}{\partial{RNAP}^{T}}Cov\left( D_{1}^{T},{RNAP}^{T} \right)+2\frac{\partial M_{2}}{\partial D_{2}^{T}}\frac{\partial M_{2}}{\partial{RNAP}^{T}}Cov\left( D_{2}^{T},{RNAP}^{T} \right)$ | (48) |
|  | $Cov(M_{1},M_{2})=\frac{\partial M_{1}}{\partial D_{1}^{T}}\frac{\partial M_{2}}{\partial D_{1}^{T}}Var\left( D_{1}^{T} \right)+\frac{\partial M_{1}}{\partial D_{2}^{T}}\frac{\partial M_{2}}{\partial D_{2}^{T}}Var\left( D_{2}^{T} \right)+\frac{\partial M_{1}}{\partial{RNAP}^{T}}\frac{\partial M_{2}}{\partial{RNAP}^{T}}Var({RNAP}^{T})+\left( \frac{\partial M_{1}}{\partial D_{1}^{T}}\frac{\partial M_{2}}{\partial D_{2}^{T}}+\frac{\partial M_{2}}{\partial D_{1}^{T}}\frac{\partial M_{1}}{\partial D_{2}^{T}} \right)Cov\left( D_{1}^{T},D_{2}^{T} \right)+\left( \frac{\partial M_{1}}{\partial D_{1}^{T}}\frac{\partial M_{2}}{\partial{RNAP}^{T}}+\frac{\partial M_{2}}{\partial D_{1}^{T}}\frac{\partial M_{1}}{\partial{RNAP}^{T}} \right)Cov\left( D_{1}^{T},{RNAP}^{T} \right)+\left( \frac{\partial M_{2}}{\partial{RNAP}^{T}}\frac{\partial M_{1}}{\partial D_{2}^{T}}+\frac{\partial M_{1}}{\partial{RNAP}^{T}}\frac{\partial M_{2}}{\partial D_{2}^{T}} \right)Cov\left( D_{2}^{T},{RNAP}^{T} \right)$ | (49) |

The simulations of the transcriptional resource competition effect and the correlations between mRNAs are shown in S3 Fig. Unlike the translational resource competition, the dependence of mRNA on RNAP ($\frac{\partial M_{1}}{\partial{RNAP}^{T}}$and $\frac{\partial M_{2}}{\partial{RNAP}^{T}}$) is weak compared to the dependence of protein on ribosome. Furthermore, the effect of transcriptional resource competition is weak ($\frac{\partial M_{2}}{\partial D_{1}^{T}}$ is close to zero) as shown in S3B Fig. Due to these parameter differences, increasing $Var\left( D_{1}^{T} \right)$ rarely changes the sign of $Cov(M_{1},M_{2})$ from positive to negative, as $Cov(P_{1},P_{2})$ changes in the Simpson’s paradox during translational resource competition.

# Note 3. Antibiotic resistance model

In the antibiotic resistance model, deactivation of antibiotics by their corresponding metabolizing enzymes was considered to be the only resistance mechanism. Other antibiotic resistance mechanisms can be modelled with similar methods. We assume that the antibiotic influx is diffusion limited, with a diffusion coefficient of $D_{A}$, and that the antibiotic deactivation follows Michaelis-Menten kinetics, with parameters $k_{cat}$ and $K_{M}$. Let $A_{ex}$ and $A_{in}$ denote the external and internal antibiotic concentrations, respectively, and let $R$ represent the expression level of resistance genes. Then

|  | $\frac{dA_{in}}{dt}=D_{A}\left( A_{ex}-A_{in} \right)-\frac{k_{cat}\cdot R{\cdot A}_{in}}{A_{in}+K_{M}}.$ | (50) |
| --- | --- | --- |

Solving for the steady state of (50), we obtain

|  | ${A_{in}}^{2}+\left( K_{M}+\frac{k_{cat}\cdot R}{D_{A}} -A_{ex} \right)A_{in}-A_{ex}K_{M}=0.$ | (51) |
| --- | --- | --- |

Equation (51) is a quadratic form of $A_{in}$, with the solution

|  | $A_{in}=\frac{1}{2}\left( A_{ex}-K_{M}-\frac{k_{cat}\cdot R}{D_{A}}+\sqrt{\left( K_{M}+\frac{k_{cat}\cdot R}{D_{A}} -A_{ex} \right)^{2}+4A_{ex}K_{M}} \right).$ | (52) |
| --- | --- | --- |

The probability that a cell can survive under an external antibiotic concentration $\delta$ is defined as

|  | $P\left( Survive \vert A_{\mathrm{ex}}=\delta\right)=P\left( A_{in}<\theta\vert A_{\mathrm{ex}}=\delta\right),$ | (53) |
| --- | --- | --- |

where $\theta$ is the highest antibiotic concentration that the bacteria can tolerate, and $A_{in}$ is a function of $R$.

This model is then extended to multi-drug resistance (Fig 5A). Drug-drug interactions, such as epistasis and cross-resistance [3], are neglected. The survival rate (probability) from simultaneous treatment with two antibiotics equals

|  | $P\left( Survive \vert A_{\mathrm{ex}1}=\delta_{1},A_{\mathrm{ex}2}=\delta_{2} \right)=P\left( A_{in1}<\theta_{1} \vert A_{\mathrm{ex}1}=\delta_{1} \right) P\left( A_{in2}<\theta_{2} \vert A_{\mathrm{ex},2}=\delta_{2} \right).$ | (54) |
| --- | --- | --- |

The joint distributions of $R_{1}$ and $R_{2}$ were then simulated using correlated gamma distributions (Fig 5B). We varied the correlation between $R_{1}$ and $R_{2}$ and fixed their means and variances in the population. The survival rates at various combinations of antibiotic concentrations were calculated based on the joint distributions. For simplicity, we used the same $D_{A}$, $k_{cat}$, $K_{M}$, and $\theta$ values for both antibiotics. We simulated the population survival from two antibiotics at various combinations of concentrations under different joint distributions of the two resistance proteins. The results show that the survival rate increases with the correlation between the resistance proteins in the presence of both A_1_ and A_2_ (Fig 5 C and D).

# Table A. Default parameters used in simulations.

| Parameter | Description | Value | Unit |
| --- | --- | --- | --- |
| ${RNAP}^{T}$ | total RNA polymerases | 4000-12000 ^a^ | copy number per cell |
| $D_{1}^{T}$ | total heterologous genes | 0-500 ^b^ | copy number per cell |
| $D_{2}^{T}$ | total endogenous genes | 700 ^c^ | copy number per cell |
| $D_{3}^{T}$ | endogenous tRNA and rRNA coding genes | 100 ^d^ | copy number per cell |
| $\alpha_{1}^{+}$ | rate constant for the formation of heterologous gene transcribing complexes | 1/10 ^a^ | s^-1^ |
| $\alpha_{1}^{-}$ | rate constant for the formation of heterologous mRNAs from their transcribing complex | 60/5000 ^e^ | s^-1^ |
| $\alpha_{2}^{+}$ | rate constant for the formation of endogenous gene transcribing complexes | 1/10 ^a^ | inverse seconds |
| $\alpha_{2}^{-}$ | rate constant for the formation of endogenous mRNAs from their transcribing complex | 60/1000 ^e^ | inverse seconds |
| $\alpha_{3}^{+}$ | rate constant for the formation of tRNA and rRNA transcribing complexes | 1/5 ^a^ | inverse seconds |
| $\alpha_{3}^{-}$ | rate constant for the formation of tRNA and rRNA from their transcribing complex | 90/5000 ^e^ | inverse seconds |
| $\nu_{1}$ | average number of RNAPs transcribing a heterologous gene | 8 ^f^ | copy number per gene |
| $\nu_{2}$ | average number of RNAPs transcribing an endogenous gene | 1.6 ^f^ | copy number per gene |
| $\nu_{3}$ | average number of RNAPs transcribing a tRNA or a rRNA gene | 11 ^f^ | copy number per gene |
| $\gamma_{1}$ | degradation rate of heterologous mRNA | log2/240 ^a^ | inverse seconds |
| $\gamma_{2}$ | degradation rate of endogenous mRNA | log2/240 ^a^ | inverse seconds |
| $\gamma_{3}$ | dilution rate for endogenous tRNA and rRNA | log2/5400 ^g^ | inverse seconds |
| ${Rib}^{T}$ | total ribosomes | 7000-15000 ^a^ | copy number per cell |
| $M_{1}^{T}$ | total heterologous mRNAs | 0-500 ^h^ | copy number per cell |
| $M_{2}^{T}$ | total endogenous mRNAs | 4000 ^a^ | copy number per cell |
| $\beta_{1}^{+}$ | rate constant for the formation of translational complex involving heterologous mRNAs | 0.002 ^a^ | inverse seconds |
| $\beta_{1}^{-}$ | rate constant for the production of heterologous proteins from their translational complex | 20/1500 ^e^ | inverse seconds |
| $\beta_{2}^{+}$ | rate constant for the formation of translational complex involving endogenous genes | 0.0002 ^a^ | inverse seconds |
| $\beta_{2}^{-}$ | rate constant for the production of endogenous proteins from their translational complex | 20/300 ^e^ | inverse seconds |
| $n_{1}$ | average number of ribosomes translating a heterologous mRNA | 30 ^f^ | copy number per mRNA |
| $n_{2}$ | average number of ribosomes translating an endogenous mRNA | 2 ^f^ | copy number per mRNA |
| $\lambda$ | growth rate | log2/5400 ^i^ | inverse seconds |
| $D_{A}$ | diffusion coefficient | 1.5 ^j^ | inverse seconds |
| $k_{cat}$ | turnover rate | 60 ^j^ | inverse seconds |
| $K_{M}$ | Michaelis constant | 12 ^j^ | μm |
| $\theta$ | tolerance | 10 ^j^ | μm |

^a^, estimated based on Bionumbers Database.

^b^, estimated based on common plasmid copy numbers.

^c^, estimated based on reference [4].

^d^, estimated based on 86 tRNA genes and 7 rRNA genes in *E. coli*.

^e^, assuming a translational rate of 20 aa/sec and a transcriptional rate of 60 nt/sec, heterologous genes with an average length of 5 kb and 1500 codons, and endogenous genes with an average length of 1 kb and 300 codons. The formation rates of all complexes were estimated assuming strong initiation rates for both transcription and translation of heterologous genes and moderate initiation rates for both transcription and translation of endogenous genes.

^f^, assuming longer genes with high transcription initiation rates have more RNAPs transcribing the genes, and longer mRNAs with high translation initiation rates have more ribosomes translating mRNAs.

^g^, approximated by growth/dilution rate, due to the stable structures of tRNA and rRNA.

^h^, estimated based on references [5,6].

^i^, based on a doubling time of 90 min.

^j^, adopted from a previous chloramphenicol-resistance model [7].

# Supplementary References:

1. Gotta SL, Miller OL, French SL, French SL. rRNA transcription rate in Escherichia coli. J Bacteriol. 1991;173: 6647–9. Available: http://www.ncbi.nlm.nih.gov/pubmed/1717439

2. Taniguchi Y, Choi PJ, Li GW, Chen H, Babu M, Hearn J, et al. Quantifying E. coli proteome and transcriptome with single-molecule sensitivity in single cells. Science. 2010;329: 533–538. doi:10.1126/science.1188308

3. Michel J-B, Yeh PJ, Chait R, Moellering RC, Kishony R. Drug interactions modulate the potential for evolution of resistance. Proc Natl Acad Sci USA. 2008;105: 14918–14923. doi:10.1073/pnas.0800944105

4. Bremer H, Dennis P, Ehrenberg M. Free RNA polymerase and modeling global transcription in Escherichia coli. Biochimie. 2003;85: 597–609. doi:10.1016/S0300-9084(03)00105-6

5. So LH, Ghosh A, Zong C, Sepúlveda LA, Segev R, Golding I. General properties of transcriptional time series in Escherichia coli. Nat Genet. 2011;43: 554–560. doi:10.1038/ng.821

6. Gorochowski TE, Avcilar-Kucukgoze I, Bovenberg RAL, Roubos JA, Ignatova Z. A Minimal Model of Ribosome Allocation Dynamics Captures Trade-offs in Expression between Endogenous and Synthetic Genes. ACS Synth Biol. 2016;5: 710–720. doi:10.1021/acssynbio.6b00040

7. Deris JB, Kim M, Zhang Z, Okano H, Hermsen R, Groisman A, et al. The Innate Growth Bistability and Fitness Landscapes of Antibiotic-Resistant Bacteria. Science. 2013;342: 1237435–1237435. doi:10.1126/science.1237435
