# Supplementary figures and images for: Heterogeneity coordinates bacterial multi-gene expression in single cells
Source: PLoS Comput Biol. 2020 Jan 31;16(1):e1007643. doi: 10.1371/journal.pcbi.1007643 (PMC7015429; doi:10.1371/journal.pcbi.1007643)

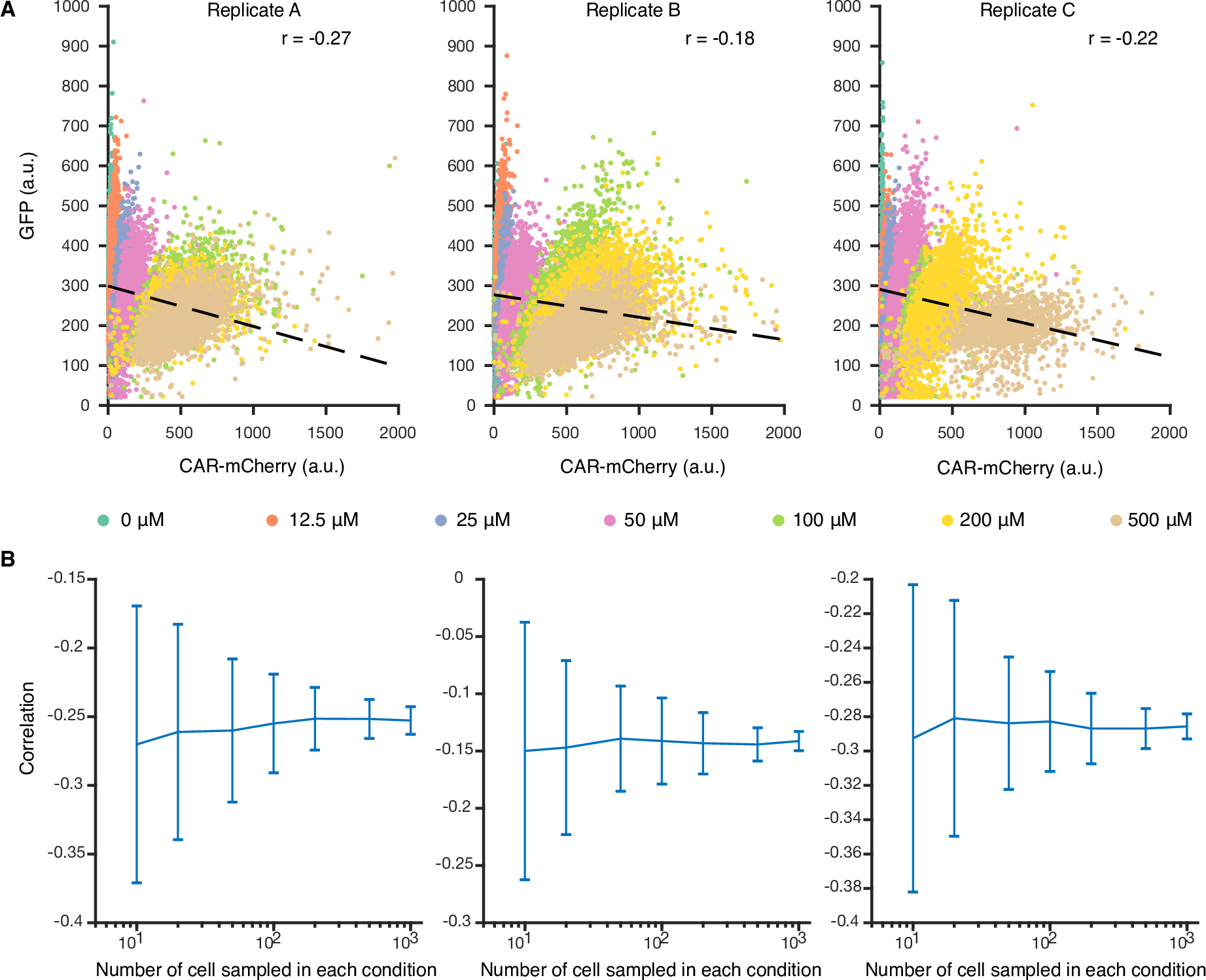

Supplement: S1 Fig — (A) Dashed lines are linear fitting of the merged data. The three replicates were performed at different days. (B) Correlation from random and evenly sampling across all induction conditions. Error bars represent standard deviations of 100 replicates. (TIF) [file pcbi.1007643.s006.tif]

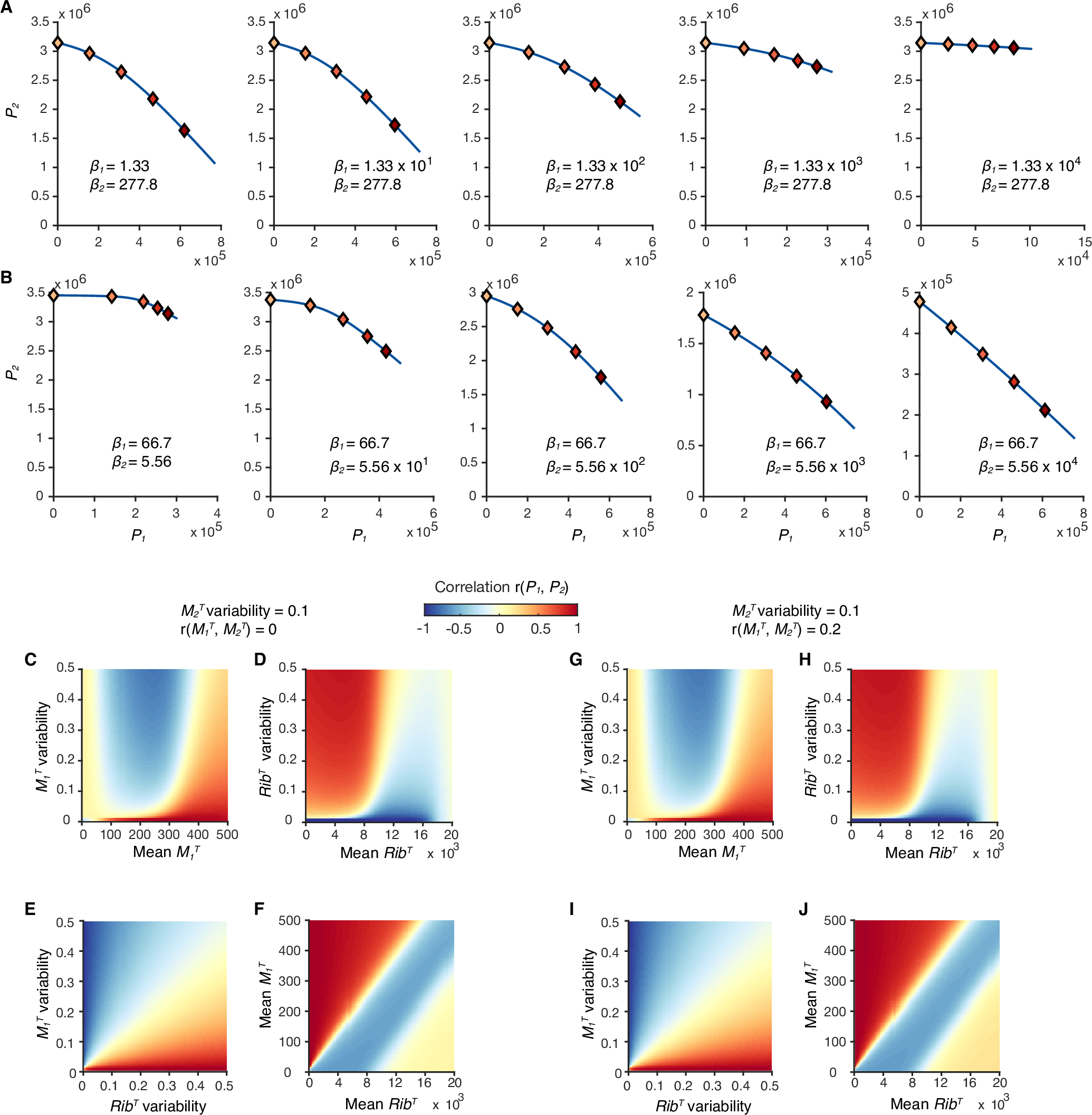

Supplement: S2 Fig — (A) The relationship between endogenous protein (P2) and heterologous proteins (P1) at various β1 values. (B) The relationship between endogenous protein (P2) and heterologous proteins (P1) at various β2 values. β1 and β2 are varied by tuning β1+ and β2+ (from 1*10−2 to 1*10−6) respectively. (C-J) The same relationship as Fig 2C–2F with M2T variability set as 0.1. (C-F) correlation between M1T and M2T is set as 0. (G-J) correlation between M1T and M2T is set as 0.2. (TIF) [file pcbi.1007643.s007.tif]

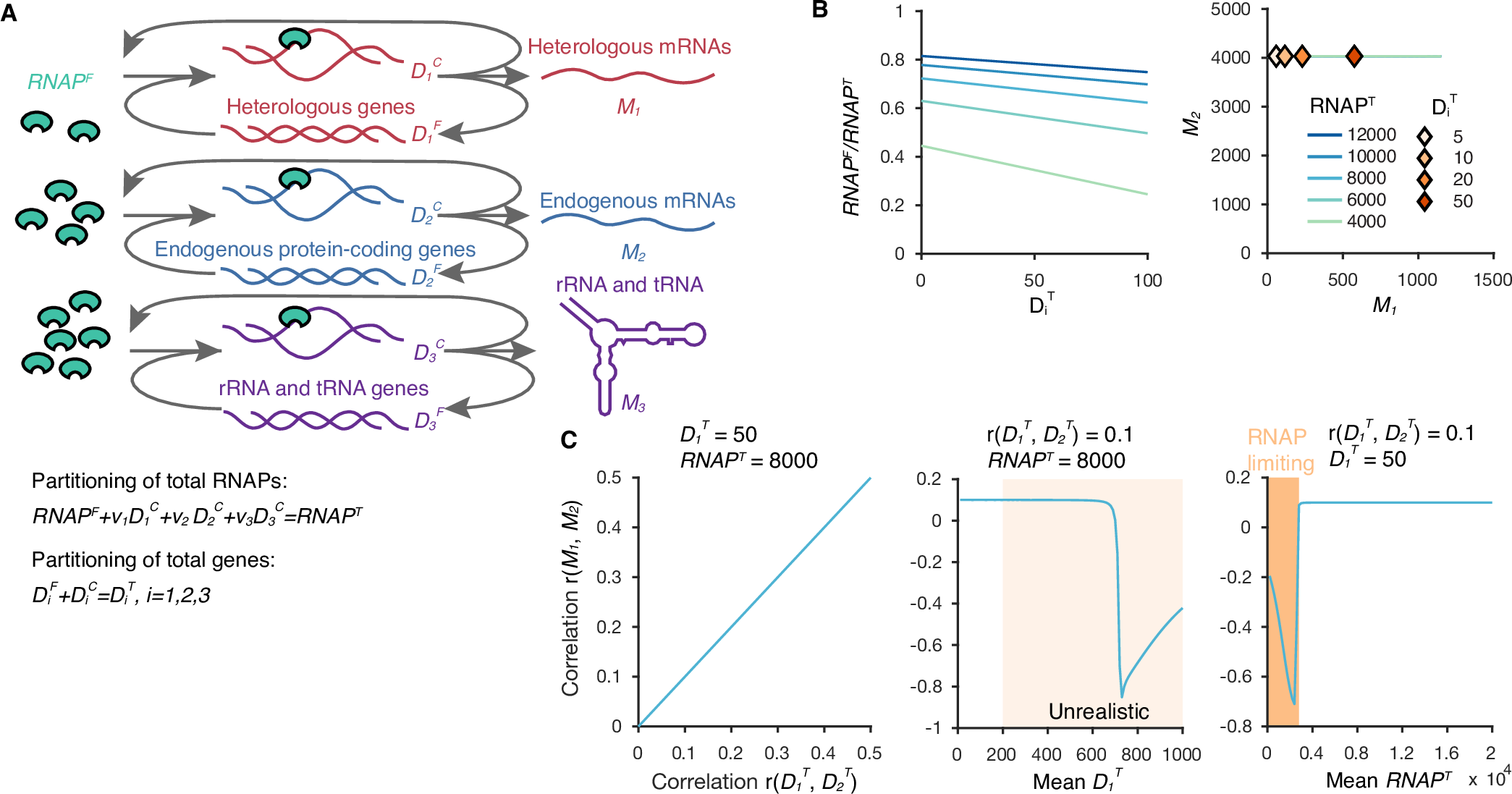

Supplement: S3 Fig — (A) Schematic of RNAP allocation among heterologous genes (i = 1), endogenous protein-coding genes (i = 2), and rRNA/tRNA genes (i = 3). RNAPF, free RNAPs; RNAPT, total RNAPs; DiF, genes free from RNAPs; DiC, gene-RNAP complexes; DiT, total genes; Mi, total mRNAs. (B) RNAP competition in a single cell. Left, relationship between D1T and the fraction of free RNAP (RNAPF/RNAPT). Right, relationship between heterologous mRNA (M1) and endogenous mRNA (M2) caused. Calculations of RNAPF, M1, and M2 are described in Note 2.2 in S1 Text with parameters listed in Table A in S1 Text. (C) Correlations between competing mRNAs in single cells r(M1, M2) changes with correlations between promoter strengths r(D1T, D2T) (left), D1T (center), and RNAPT (right). D1T > 200 is considered as unrealistic region. RNAPT affects r(M1, M2) only in RNAP limiting region. (TIF) [file pcbi.1007643.s008.tif]

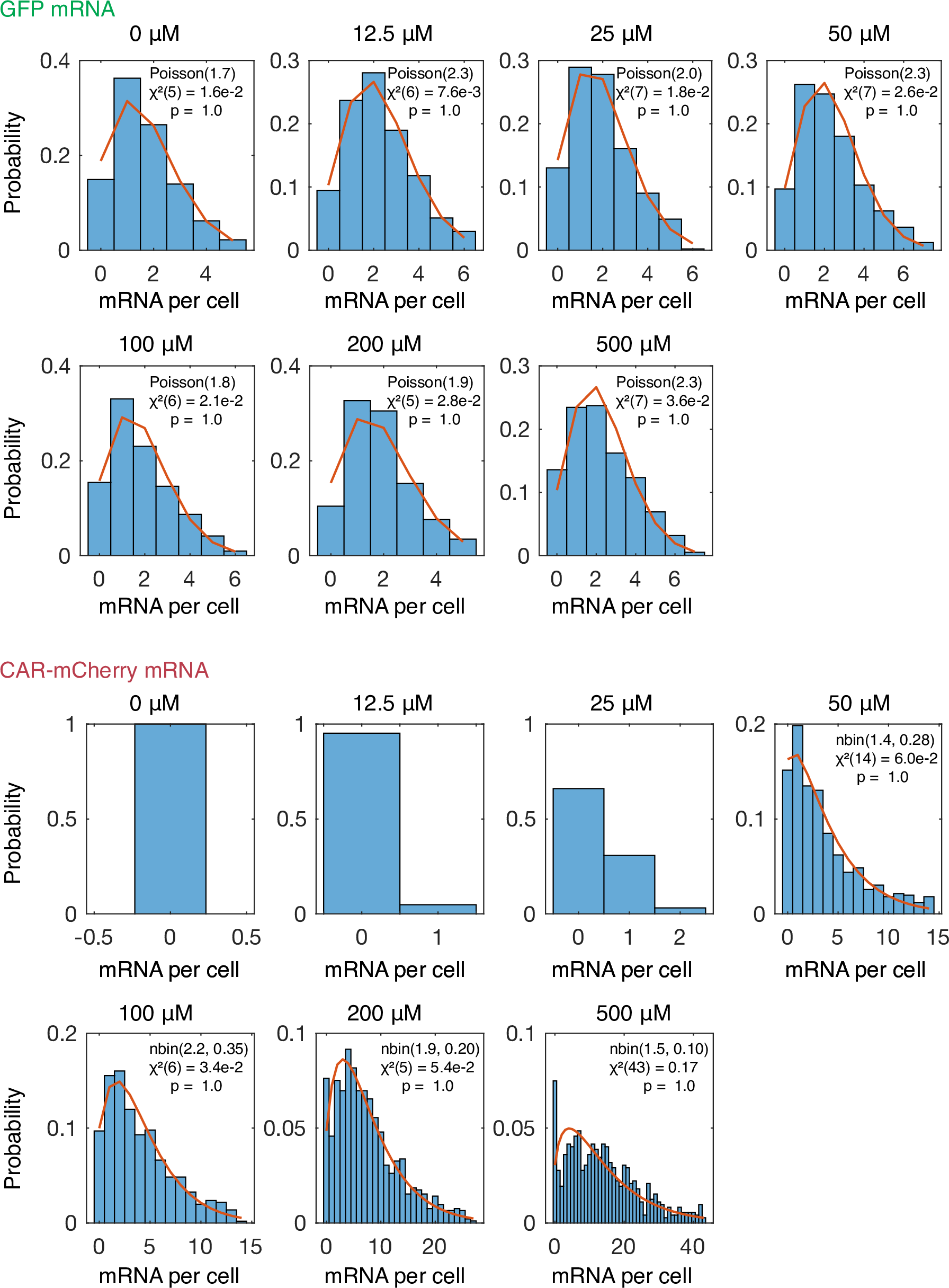

Supplement: S4 Fig — Single-cell GFP mRNA copy numbers measured from FISH were fitted to Poisson distributions due to its transcription from a constitutive promoter. CAR-mCherry mRNA copy numbers were fitted with negative binomial distributions because they were transcribed from an inducible promoter. (TIF) [file pcbi.1007643.s009.tif]
