# Supplementary material for: Heterogeneity coordinates bacterial multi-gene expression in single cells
Source: PLoS Comput Biol. 2020 Jan 31;16(1):e1007643. doi: 10.1371/journal.pcbi.1007643 (PMC7015429; doi:10.1371/journal.pcbi.1007643)
Supplement: Attachment — Submitted filename: Response to reviewers__final.DOCX [file pcbi.1007643.s010.DOCX]

**Comments to the Authors:**

Reviewer #1: The article presents experimental and modeling work on the correlation between protein levels (and between RNA levels) in a population of genetically identical bacteria. The central finding concerns the relationship between the anti-correlation of protein levels that occurs when different conditions are merged and the positive correlation that characterizes each condition individually. Fluctuations in available resources (mRNAs in particular) are identified as the key to explain observations. On the plus side I found the paper quite well written. The modelling results and experimental data are presented clearly and thoroughly. On the minus side, the modeling framework needs to be placed in the context of existing models, and the interpretation of the main finding is in my view not completely convincing.

Main:

1) The general problem faced in this work (competition, allocation of cellular resources etc.) is well studied and the model discussed in this paper seems to be close to previous models both technically and in terms of the questions being asked and lessons being drawn (the fact that P1 is considered as “heterologous” seems to me to be immaterial for the conclusions). For instance, the fact that (the expression levels of) two proteins display negative correlation (in any given condition) immediately suggests, as the authors say, competition for shared positive regulators (eg ribosomes). The full picture is however much richer and includes the possibility of having positive correlations, depending essentially on kinetic details (see e.g. 10.1016/j.bpj.2013.04.049 & [doi.org/10.1371/journal.pcbi.1002203](http://doi.org/10.1371/journal.pcbi.1002203)). On the other hand a positive correlation can also be induced by competition for a shared negative regulator of gene expression such as microRNAs (acting on mRNAs, see e.g. 10.1038/srep43673). (The suggested links only represent a few examples that came to my mind, but the modelling literature on these topics is huge.) In each case, correlations between the corresponding transcripts do not need to reflect those between their functional products. In my view, a discussion of previous approaches and of how the present model deviates from/generalizes/complements them is necessary. In particular, it would be important that authors clarify what biological insight discussed in this paper cannot be obtained without the specific modeling frame/assumptions they employed. In this respect, I think that the role of resource variability could be further highlighted against previous work.
**Answer:** We thank the reviewer for this insightful comment. As requested, we have added discussions of previous modeling approaches and the differences with our model. We further highlighted the consideration of resource variation in our model as a novel component in this study.

Specifically, our work differentiates from previous papers as we focused on resource variability. In our model, the amounts of resource available for gene expression, such as RNA polymerase or ribosome, are allowed to vary among single cells. Previous models, except for the one studied by Mather *et al* [20], did not consider this resource variability, thus gene expression properties at the single-cell level under competition conditions were unclear. Although Mather *et al* mentioned ribosomal fluctuation, sources of correlation were never analyzed.

Furthermore, our model was built to understand competition between the heterologous expression system and the native expression system, which was seldom considered in previous models. Modeling heterologous expression systems is important and unique for the following reasons. 1) Heterologous expression systems have direct applications in recombinant protein production, metabolic engineering, and other engineering projects. 2) Our heterologous expression system has the advantage of well-defined regulation, which rules out effects from the native regulatory system such as transcription factors and small RNAs as other papers suggested. This feature allows us to focus on the generic resources in transcription and translation such as ribosome, RNAP. 3). Heterologous expression system in engineering settings is often more burdensome than expression from native system. Thus, the resource competition effects in heterologous systems are much stronger than that from native systems as studied in previous papers.

We have revised the paper accordingly. Please see lines 115-124.

2) Regarding the Simpson paradox, merging data coming from different conditions does not necessarily yield, as far as I understand, a new condition from which conclusions can be drawn. In general I would say that cases like the experiment of Hecht & al [S Hecht, S Shlaer, and MH Pirenne, Energy, quanta and vision. , J Gen Physiol 25, 819–840 (1942)] provide a strong caveat against doing it: averaging over different conditions (patients in their case) can lead to erroneous interpretations of data. I understand that the authors take the merged dataset to model a “heterogeneous and fluctuating” environment, but frankly I am not convinced. Looking at individual conditions one would conclude that the competition between the two proteins is not there and everything is driven by the induction that changes the slope of the protein-protein dependence across different conditions. The fact that mixing experiments one observes a negative correlation does not change this fact. So why exactly do authors deem it important/interesting that averaging over conditions the correlation changes sign? Why is this special? This is really not clear to me, also because the negative correlation of mean values seems to be rather weak (especially when compared against the range of variability of single cells).

**Answer:** We thank the reviewer for this comment. Based on our analysis, the positive correlation is caused by the relatively small variation of mRNA expression level under each condition, while merging dataset from different conditions leads to large variations in mRNA, thus resulting in negative correlation. This merging data from different conditions mimic the fluctuating environment in multiple systems and has realistic meanings/applications. For example, in biofilm, large chemical gradients often exist and can cause large variation in mRNA level for certain genes (<https://www.nature.com/articles/nrmicro1838>), while mRNA variation for cells within a small region of that biofilm could be small. If bacteria are collected from a small region of a biofilm, positive correlation between two proteins is expected, representing a form of “multitasking”. However, cells collected from the whole biofilm may exhibit negative correlation between the two proteins, representing a mode of “division of labor”. Thus, the Simpson’s paradox phenomenon is important to understand bacterial population behavior in biofilms. Additionally, Simpson’s paradox phenomena can affect multiple engineering systems. For example, in large-scale fermentation, large fluctuation in nutrient condition within a fermenter coexists with well mixed local regions. Thus, our model will be found useful in analyzing population performance and in microbial design to improve overall yield (https://www.nature.com/articles/nchembio.2046).

We have revised our manuscript to better explain the importance of the studied Simpson’s paradox (line 92-95)

3) Important: it seems to me (Table S3) that the nr of cells is rather unevenly distributed across induction conditions (less cells at the maximum level compared to the control). Am I right in assuming that the fitting procedure used for the merged data accounts for this imbalance? (Otherwise the fit could be biased to return a negative correlation). This should be made very clear all throughout the text.
**Answer:** We thank the reviewer for pointing this out. The number of cells sampled in each condition could affect/bias the exact value of correlation coefficient. In our case, a large number of cells were quantified (n>1000) to rule out this potential effect from unevenly sampled data. The data still represent the natural scenario where cells live in a highly heterogeneous environment.

To further prove, we took the reviewer’s suggestion by performing random and evenly sampling across all induction conditions. Different sample sizes from 10 to 1,000 were used. A total of 100 replicates of the sampling process were performed to estimate the standard deviations of correlation. The results clearly showed that the negative correlations are not affected by sample size and the standard deviation of correlation decreases with larger sample size (Fig. S1B).

We have added this analysis to the main text after the conclusion of negative correlation (line 87-89).

Figure legend: Correlation from random and evenly sampling across all induction conditions. Error bars represent standard deviations of 100 replicates.

Minor:

1). Supplementary Note: The first equation (unnumbered) of Note 1 as well as the first equation (unnumbered) of Note 2 appear on a single line without any separation in my doc reader. This is confusing (but it may depend on the reader, not sure)
**Answer:** We have added these two equations into the numbered equation list.

2). About parameters: Models tend to use somewhat standardized parameters, so I don’t doubt that the representative results displayed by the authors represent a physiologically realistic scenario. However a discussion of how sensitive results are to the model’s parameters would be welcome.

**Answer:** We thank the reviewer for this suggestion. We ran the model under a wide range of parameters and conclusions we made are robust.

In translational resource competition model, in order to have the competition effects, *β_1­_* cannot be much larger than *Rib^F^* and *β_2­_* is much smaller than *Rib^F^*. We have discussed the reason in the main text in the revised version (lines 134 to 139).

The correlations between competing proteins *r(P_1_*, *P_2_*) are mainly determined by mean value and variance of *M_1_^T^*and *Rib^T^*, and are slightly affected by variance of *M_2_^T^* or correlation between *M_1_^T^* and *M_2_^T^* when *M_1_^T^* is low and *Rib^T^* is high (Fig. S2 C to J). However, the correlations between competing mRNA *r(M_1_*, *M_2_*) are mainly determined by correlation between *D_1_^T^* and *D_2_^T^* (Fig. S3 x). The reason is because the dissociation constants in the transcriptional resource competition model, *α_1­_* and *α_2_*, are much smaller than the amount of free RNAP (i.e. competition effects are weak). We have added these discussions in the revised version (line 181-182, 194-195, Fig S2 and S3 in the revised version)

3). Equation 1 plays a central role in this manuscript. The approximation based on which it is derived (Supplementary Note 1) seems reasonable to me but I would stress it in the Main Text. Also, Eq 1 is rather intuitive once the approximation is explained. I suggest the authors provide the reader with some guideline to interpret the physical meaning of Eq 1 already in the Main Text.
**Answer:** We have added more explanations of the key modeling results to the main text (line 128-139, 154-158).

Reviewer #2: The work by Han and Zhang reports an extremely interesting study on resource competition in single cells at the translational and transcriptional level. The authors found that the former correlates positively in single cells, but negatively at in the population. This Simpson paradox is not found at the transcriptional level.

The work is very nicely and concisely summarized. I only have some minor suggestions
**Answer:** We thank the reviewer for the positive evaluation of our manuscript.

1). as the authors submitted to PLOS CB, I do not think the mathematical model needs to be hidden in the supplementary materials. Rather the model and major mathematical results should be explicitly shown in the main text. I believe this is particularly true for line 80 to 130, where the train of thought is interrupted by reference to the supplementary material.

**Answer:** We have added the key modeling components to the main text. We further described how the key parameters affect the modeling results (line 128-139, 154-158).

2). the authors say within certain ranges (line 107) with feasible parameters (line 116-117). I think these number should be made explicit (including some discussion) in the main text.

**Answer:** We have explicitly added all those parameter values in the main text (line 179, 190-192).

3). the connection between mathematical model and experimental realization may be improved if Fig 1A for instance also includes the model variables.

**Answer:** We thank the reviewer for this suggestion. To improve the connection, we have moved the original Fig S2, which includes all the model variables, to the main text as new Fig.2. We prefer to keep the original Fig 1A because Fig. 1 shows the experimental results. The modeling variables are now shown in Fig 2, right after experimental results.

4). the authors say shining light on a previously debated issue (line 131-132). Please, could you briefly indicate the arguments put forward by the references in this debate.

**Answer:** Results from Gyorgy et al. [7] indicate that RNA polymerases are a limiting resource that can contribute to burden. Their study did not directly measure RNA, and the evidence is based on protein measurements and two important model assumptions: 1) RBS sequence only affect translation initiation rate; 2) To reach the same protein concentration, weaker RBS requires more mRNA, thus an increased usage of RNAP, and the same usage of ribosomes. Both assumptions could fail, e.g., RBS affects RNA lifetime or weaker RBS requires different amounts of ribosomes to obtain the same protein concentration.

Ceroni et al. [29] performed RNA-seq and showed that effects on transcription are a major factor in burden. The data is based on normalized read counts and they did not have spike-in control standards. So the role of transcription burden can be overestimated by the normalization steps.

Recently, a more comprehensive study by Cambray et al. [30] concluded that overexpressing mRNA causes burden by sequestering ribosome rather than by making or maintaining transcripts. This is consistent with our direct RNA measurement.

These papers have been cited in the manuscript. We further added “debated issue about the cause of mRNA burden” to specify the debate (line 208).

5). line 134 - 136, what would be required to reverse the correlation so that’s consistent with line 119?

**Answer:** The correlations between competing mRNAs are determined by correlations between promoter strengths, and the promoter strength correlations can be weak or even negative in constant environments. If the correlation between promoter strengths is set to zero (based on the independence assumption) in the model, the mRNA correlation could be negative. However, correlation between promoter strengths in our experimental setup is larger than 0.1, likely due to cell division. So mRNA correlation is also positive in the experiment. Based on our modeling analysis, the mRNA correlations rarely change with promoter strength and its variability. With the default parameters we used, the mRNA correlation gets affected by promoter strength only when we have more than 700 copies of strong promoter in the heterologous system, which is very rare in reality.

We have clarified the role of promoter strength correlations in our revised version (line 194-195, 210-211). We also edited the original Fig S3C to highlight the effect of promoter strength correlations.

Figure legend: Correlations between competing mRNAs in single cells r(*M_1_*, *M_2_*) changes with correlations between promoter strengths r(*D_1_^T^*, *D_2_^T^*) (left), *D_1_^T^* (center), and *RNAP^T^* (right). *D_1_^T^* > 200 is considered as unrealistic region. *RNAP^T^* affects r(*M_1_*, *M_2_*) only in RNAP limiting region.

6). please deposit your data and matlab scripts on github or some other public database
**Answer:** We thank the reviewer for this suggestion. The raw data and MATLAB scripts have been deposited on GitHub: https://github.com/yhan0410/Data-and-model-in-Heterogeneity-coordinates-bacterial-multi-gene-expression-in-single-cells.
